# Simulations for designing and interpreting intervention trials in infectious diseases

**DOI:** 10.1186/s12916-017-0985-3

**Published:** 2017-12-29

**Authors:** M. Elizabeth Halloran, Kari Auranen, Sarah Baird, Nicole E. Basta, Steven E. Bellan, Ron Brookmeyer, Ben S. Cooper, Victor DeGruttola, James P. Hughes, Justin Lessler, Eric T. Lofgren, Ira M. Longini, Jukka-Pekka Onnela, Berk Özler, George R. Seage, Thomas A. Smith, Alessandro Vespignani, Emilia Vynnycky, Marc Lipsitch

**Affiliations:** 1 Vaccine and Infectious Disease Division, Fred Hutchinson Research Center, 1100 Fairview Ave N, Seattle, WA 98109 USA; 2 Department of Biostatistics, School of Public Health, University of Washington, Seattle, WA USA; 3 Department of Mathematics and Statistics, University of Turku, Turku, Finland; 4 Department of Global Health, Milken Institute School of Public Health, The George Washington University, Washington DC, USA; 5 Division of Epidemiology and Community Health, School of Public Health, University of Minnesota, Minneapolis, MN USA; 6 Department of Epidemiology and Biostatistics, College of Public Health, University of Georgia, Athens, GA USA; 7 Department of Biostatistics, The Fielding School of Public Health, UCLA, Los Angeles, CA USA; 8 Mahidol Oxford Tropical Medicine Research Unit, Bangkok, Thailand; 9 Department of Biostatistics, Harvard T.H. Chan School of Public Health, Boston, MA USA; 10 Department of Epidemiology, Johns Hopkins Bloomberg School of Public Health, Baltimore, MD USA; 11 Paul G. Allen School for Global Animal Health, Washington State University, Pullman, WA USA; 12 Department of Biostatistics, University of Florida, Gainesville, FL USA; 13 Development Research Group, The World Bank, Washington DC, USA; 14 Department of Epidemiology, Harvard T.H. Chan School of Public Health, Boston, MA USA; 15 Department of Epidemiology and Public Health, Swiss Tropical & Public Health Institute, Basel, Switzerland; 16 University of Basel, Basel, Switzerland; 17 Network Science Institute, Northeastern University, Boston, MA USA; 18 Modelling and Economics Unit, Public Health England, Colindale, UK; 19 TB Modelling Group, Centre for Mathematical Modelling of Infectious Diseases, TB Centre and Faculty of Epidemiology and Population Health, London School of Hygiene and Tropical Medicine, London, UK

**Keywords:** Clinical trial design, Infectious diseases, Mathematical modeling, Simulations, Vaccine

## Abstract

**Background:**

Interventions in infectious diseases can have both direct effects on individuals who receive the intervention as well as indirect effects in the population. In addition, intervention combinations can have complex interactions at the population level, which are often difficult to adequately assess with standard study designs and analytical methods.

**Discussion:**

Herein, we urge the adoption of a new paradigm for the design and interpretation of intervention trials in infectious diseases, particularly with regard to emerging infectious diseases, one that more accurately reflects the dynamics of the transmission process. In an increasingly complex world, simulations can explicitly represent transmission dynamics, which are critical for proper trial design and interpretation. Certain ethical aspects of a trial can also be quantified using simulations. Further, after a trial has been conducted, simulations can be used to explore the possible explanations for the observed effects.

**Conclusion:**

Much is to be gained through a multidisciplinary approach that builds collaborations among experts in infectious disease dynamics, epidemiology, statistical science, economics, simulation methods, and the conduct of clinical trials.

## Background

Designing intervention trials for infectious diseases poses many challenges. Firstly, for many infectious diseases, in addition to the direct effects on those receiving the intervention, interventions can have indirect effects on individuals not receiving the intervention as well as on those receiving the intervention. These indirect effects, sometimes called ‘spillover effects’, may affect the estimation of the direct effects and are also of public health significance themselves. Secondly, because transmission is a non-linear and stochastic process, outcomes in different arms of an intervention trial may be more variable than expected in a population where each individual’s outcome is statistically independent of the outcome of other individuals. Thirdly, heterogeneity from different sources, such as host susceptibility, pathogen variability, and exposure heterogeneity, can complicate study design. Finally, the effects of a combination of interventions in a trial, such as vaccination and behavioral intervention, may be difficult to predict at the design phase. Other factors, including logistical complexities and ethical considerations, can add to these challenges. Following completion of a trial, interpreting unexpected trial results can also be difficult.

Recently, investigators have used computer simulation to assist in the design, analysis, and interpretation of randomized trials of infectious disease prevention measures to address these challenges. Herein, we describe these challenges in more detail and illustrate ways in which simulation can help to conduct better trials and to improve the understanding of trial results. We conclude by advocating that, for many infectious disease prevention trials, simulating the trial with the underlying transmission dynamics is an efficient way to compare different designs and to identify key aspects critical to its success, thereby improving the choice of design.

## Challenges of designing intervention studies for infectious diseases and the role of simulations

In the design phase of randomized trials of social or biomedical interventions, investigators consider options for how to conduct the trial and ultimately choose the trial population(s), the intervention or control conditions that will occur in each trial arm, the primary and secondary outcomes to be measured, and the way in which randomization will occur. For any set of such choices, a biostatistician working on the study can estimate the range of likely outcomes that could occur in trials of various sizes, then estimate the required sample size to achieve a specified power. This estimate often comes from closed-form equations that produce accurate sample size estimates under defined assumptions about the expected frequency of the outcome in the absence and presence of the intervention, and the amount of variability expected in the outcome within and between the trial arms. For many applications outside of infectious diseases, plausible assumptions about these quantities can be directly made based on previous trials, preclinical studies, or theoretical considerations. In particular, for non-infectious diseases, disease frequencies in participants randomized to the control group can be reasonably assumed to be similar to those in groups of individuals not receiving the intervention. Frequencies in those randomized to the intervention can be assumed to be the same as those in the control group, reduced by a factor proportional to coverage and adherence of the intervention times the efficacy of the intervention. However, in infectious diseases, these assumptions are often violated due to the indirect effects of the intervention.

### Complications for direct effects

**Fig. 1:**
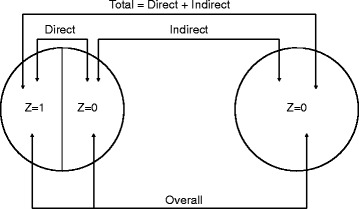
Study designs for dependent happenings. Two clusters, or populations, are considered under two different scenarios. In the left-hand side scenario, a certain portion of individuals in the cluster receive vaccination (or other treatment) (Z = 1) and the remaining portion receive the control intervention (Z = 0). In the right-hand side scenario, everyone receives the control intervention. Control is defined as current best practice, placebo, or nothing. The direct, indirect, total, and overall effects of intervention are defined by the indicated contrasts (adapted from Halloran and Struchiner [2, 3]). The effects have recently been given alternative terms in the economics literature [4], where ‘direct effect’ is termed as ‘value of treatment’, ‘indirect effect’ as ‘spillover effect on the non-treated’, ‘overall effect’ as ‘total causal effect’, and ‘total effect’ as ‘intention-to-treat effect’

In trials of vaccines and other infectious disease prevention measures, interventions on any given trial participant may affect the risk of the outcome on others, regardless of whether or not they are participating in the trial. For example, recipients of an efficacious vaccine are less likely to become infected, but may also be less infectious if they are. Infectious diseases are an example of dependent happenings, where the frequency of the outcome depends on the number already affected, which can be changed by the intervention [1]. Figure 1 illustrates some of the different effects that might occur in infectious disease interventions [2, 3]. Consider two clusters, or populations, of individuals. In one of the populations, a certain portion of individuals is vaccinated and the rest remain unvaccinated. In the other population, no one is vaccinated. The direct effect of vaccination in the population in which some individuals were vaccinated is defined by comparing the average outcomes in vaccinated individuals with the average outcomes in unvaccinated individuals. The indirect effects are defined as a contrast between the average outcomes in unvaccinated individuals in the population with vaccination and the average outcomes of unvaccinated individuals in the unvaccinated population. The total effects are defined by comparing the average outcomes in the vaccinated individuals in the vaccinated population to the average outcomes in the unvaccinated individuals in the unvaccinated population. The overall effects are defined by the contrast in the average outcomes in the entire population where some individuals were vaccinated compared to the average outcomes of the entire population that did not receive vaccination.

In Fig. 1, we have not distinguished trial participants from non-participants. In the population in which some individuals were vaccinated (left panel, Fig. 1), some of the unvaccinated may be in the control arm of the trial and some may not be in the trial at all. The indirect effects may reduce the incidence of infection for others within or outside the trial, invalidating the simple assumption that the incidence prior to the trial will be similar to the incidence in trial participants who did not receive the intervention. For complex behavioral interventions to reduce infectious disease transmission, such as education programs designed to encourage sexual abstinence to reduce transmission of infections, those who receive the intervention and change their behavior may cause others in the population to do so too in ways that will also affect the risk infection of trial participants. Due to such effects, the mean incidence in each group may depend, in complicated ways, on the intervention and trial design, which can be addressed with simulations.

### Measuring effects beyond the individual level

While the indirect effects discussed above can complicate the design and analysis of intervention trials, measuring them may be of scientific interest beyond, or even instead of, measuring the direct effects of the intervention in protecting individual recipients. Establishing that vaccination provides population-level effects that go beyond the direct effects in the vaccinated can have important consequences for public health policy. Some interventions, such as treatment-as-prevention of HIV and transmission-blocking malaria vaccines, have indirect effects alone. The overall effect of an intervention is often the quantity of greatest interest for policy-makers (Fig. 1), as it summarizes the public health consequences of the choice of intervention strategy if adopted in a population [2]. Establishing that vaccination produces indirect effects in the unvaccinated can make a vaccination strategy more cost-effective. The expected size of these population-level effects depends not only on the size of the direct effect, but also on factors related to the transmission of the disease in the population and the distribution of the intervention. Thus, additional tools that account for these interactions may be required in the design of studies to measure them.

If evaluating population-level effects of interventions, such as the total, indirect or overall effects, is of interest, then a cluster-randomized study will generally be the design of choice [2]. In simple cluster-randomized studies, the clusters are randomized to intervention or control. In two-stage randomized studies, clusters are randomized to one of several possible levels of coverage, also called saturation, of the intervention (possibly zero or pure control), and subsequently individuals within the clusters are randomized to receive, or not, the intervention with a probability equal to the coverage assigned to the cluster [4, 5].

Simulations can examine the properties of different types of cluster-randomized designs, whether parallel (clusters initially randomized to intervention or control), stepped wedge (the order in which clusters receive intervention is randomized before the trial) [6], or something else. For example, the ring vaccination trial of an Ebola vaccine compared the outcomes in rings of contacts and contacts of contacts around a detected case and randomized each ring to receive either immediate or delayed vaccination [7, 8]. For each type of design, simulations can answer questions such as ‘what is the required sample size with this design, given the likely degree of transmission during the trial [9]?’, ‘what is the optimal choice of cluster size versus number of clusters?’, or ‘what are optimal coverage (saturation) levels across clusters [4]?’ Simulations can also compare different types of designs, clarifying the tradeoffs among these designs in power and bias for estimating various quantities of interest. Recently, simulations have been used to design a stepped wedge cluster-randomized study of the effectiveness of adding solar-powered mosquito trapping systems to standard malaria interventions, examining different methods of temporally introducing the intervention across an island [10].

Simulations can also help predict potentially harmful indirect effects. In an intervention where women are encouraged to refuse sexual acts with men, other women in the population, either study participants or nonparticipants might be sought out by the men refused by women in the trial; these are known as spillover effects [4] or displacement [11]. If displacement occurs, the incidence of HIV may be higher in the women not benefitting from the intervention than it would have been if the trial had not occurred. In malaria interventions, when some individuals use bednets, the individuals not using them may be bitten more often and have increased incidence of malaria. Alternatively, with insecticide-treated bednets, mosquitoes killed because one participant in the intervention arm of a trial uses a treated net, may then fail to bite a person who is in the control arm of the trial, reducing the risk in the control group, and thus reducing power. These spillover effects are part of the dynamic process that can be included in simulations and can therefore be accounted for in the design phase.

### Transmission as a cause of overdispersion

Not only the average risk, but also the variability in risk among individuals receiving a particular intervention may be hard to predict in the infectious disease context. Classical trial sample size calculations rely on simple assumptions about variability for independent events, which may be invalid because they do not account for complex social and sexual networks. Because of the dynamics of the transmission process and random events, a group, such as a village or hospital, within an arm of a trial may have considerably more or fewer cases than the average value. The amount of this variability may depend on how the trial is designed, such as whether individual persons or groups of persons are randomized to trial arms, and how the intervention is rolled out over time in the trial. In HIV prevention trials, one needs to account for stochastic variation in the spread of HIV from overlapping sexual networks and heterogeneity in biological and behavioral risk factors, in addition to the usual variability in standard study design [12]. Simulations can take into account different sources of variation as well as include sensitivity analyses to assess the effects of unknown sources of variation in calculating sample sizes.

### Combinations of interventions

Combinations of interventions in infectious diseases may have interactions at the population level that are difficult to predict or to express in simple equations. When different single interventions or combinations of interventions are being considered, simulations can be used to explore possibly synergistic effects on transmission and outcomes relevant for trial design. Boren et al. [12] used simulations to estimate the effect size expected from each of four HIV-prevention interventions if they were to be implemented individually or in varying combinations in a South African population. These effect size estimates could be the basis for sample size calculations in a cluster-randomized trial and to evaluate which interventions to test first. Here, assumptions could be made about the effect of each intervention on its own on an individual’s risk of contracting HIV, but simulations are necessary to understand how these interventions would affect transmission in the overall population, via indirect effects, as well as to understand how the effects of the different interventions would combine at the population level. In addition, the importance of this type of work will increase as partially effective interventions become adopted as standard of care and multiple interventions are layered upon each other. Studies will therefore need to become increasingly larger to be able to detect significant differences between arms, increasing the variability and complicating the interpretation and sample size estimates.

### Heterogeneities in hosts and pathogens

The above complexities of trial design can be compounded by additional sources of heterogeneity. Individuals may differ dramatically in both their exposure to infection and their responsiveness to the intervention. Trial planners may have little advance knowledge about the distribution of these sources of variability. Additionally, interactions between different pathogen strains can create further variability in outcome risk, which is difficult to incorporate into simple equations. For example, in the case of pneumococcal carriage, nasopharyngeal carriage of one strain in an individual inhibits colonization by another strain. A pneumococcal vaccine protects against colonization by some but not all strains of the pathogen. Therefore, if individuals acquire a non-vaccine strain, they have additional protection against acquiring another strain, including the vaccine strains. This competition could potentially make a vaccine seem either more or less efficacious than it is [13]. Furthermore, epidemics of infectious diseases typically undergo an initial phase of expansion, during which more and more people become infected, increasing the risk to others, and eventually a subsequent phase of contraction as previously susceptible people become infected and immune, decreasing the risk to others. Thus, the inputs for sample size calculations are a moving target in infectious diseases, sometimes greatly varying both temporally and spatially. Occasionally, the actual quantities that can be measured from the data observed in a trial are non-linear functions of the biological efficacy of a vaccine or drug [13–15], which may be the quantity of most direct interest or that considered most likely to be transportable to populations beyond where the trial was conducted. Simulations can help capture the impact of heterogeneity in the trial population on power and on the size of the effect being measured. For example, heterogeneity in the exposure or susceptibility to infection of trial participants can bias vaccine efficacy estimates toward the null. However, simulations showed that, under certain assumptions, these biases can be avoided by accounting for variation in frailty in the analysis [16]. Simulations considering strain-to-strain interactions have demonstrated that, even if estimation of heterogeneous protection of each component against pneumococcal carriage fails, unbiased and interpretable estimation of summary measures of vaccine efficacy may still be possible from the observed data [17].

### Improving trial logistics and ethics

Simulations can help identify key elements beyond the choice of sample size and outcome measures that determine the potential for success of a specific trial, including speed of case ascertainment, test results, or other time-sensitive processes that affect the likelihood of success. Ethical or logistical reasons may motivate the choice of design, particularly for trials in the emerging infectious disease or outbreak settings. The debate over trials of Ebola vaccines during the 2014–2015 epidemic in West Africa illustrates that, especially in emergency situations, these considerations may place competing pressures on study design [18]. For example, in the Ebola ring vaccination trial in Guinea, in the face of declining transmission, if cases had not been ascertained quickly enough and the contacts of the cases and the contacts of contacts had not been found in a timely fashion, then the trial likely would not have been feasible. The ring vaccination cluster-randomized trial ultimately demonstrated the effectiveness of one Ebola vaccine [7, 8]. The lesson was learned from the Ebola experience that intervention trials must be targeted where the transmission is or where it is expected to be. If vaccination had been allocated randomly in the population, the trial would not have had sufficient power for a conclusive result. Similarly, simulations are currently being used to help identify potential sites and study designs for Zika vaccine trials.

Besides being good scientific practice, designing a trial with adequate power is also an ethical requirement. Underpowered studies create a burden on participants without providing a high degree of assurance that the scientific results will be valuable [19], while unduly large studies place a burden on too many participants without significant extra benefits from the larger sample size. Further, poorly considered design runs the risk of discarding interventions that might be useful. Simulations can help find the design that is optimal in terms of either power, speed, or number of deaths/cases averted, and that also fulfills requirements for the respect of human dignity and other ethical tenets. For example, a cluster-randomized stepped wedge trial was proposed to vaccinate frontline healthcare workers with a candidate Ebola vaccine as quickly as logistically feasible, whilst randomizing the order in which each treatment unit received vaccination to allow randomized evaluation of vaccine efficacy. However, simulations found that an individually randomized controlled trial that prioritized the highest risk treatment units first would have greater statistical power and speed to a definitive result, avoiding more total healthcare personnel infections than the proposed stepped wedge design [20]. This kind of work has a long history in cancer treatment trials [21], but has been used less in infectious diseases [22, 23]. The expected value of the information from a trial for decision-makers relative to its cost can also be easily estimated using simulation models with an economic component [24]. In the future, interactions among trial designers, epidemiologists, infectious disease modelers, and ethicists may help to specify ethical desiderata for trials and the designs best suited to accomplish these while maintaining ethical treatment of individuals and study validity.

### Interpreting results of a trial

After a trial, simulations can be helpful in interpreting puzzling or unexpected trial results. A cluster-randomized trial was conducted to compare the effectiveness of isoniazid preventive therapy given on a community-wide basis to that of standard of care on tuberculosis (TB) in gold miners in South Africa [25]. Although pre-trial mathematical modeling had suggested that the intervention had unusually high potential for TB control [26], the intervention trial demonstrated no effectiveness on TB, attributed to lower than expected uptake. Post-trial modeling took advantage of data from the trial and demonstrated that, even with optimal uptake, a combination of interventions would be required to greatly reduce TB incidence [27]. Similarly, trials assessing the effect of sexually transmitted disease treatment on HIV incidence in Rakai, Uganda, and Mwanza, Tanzania, had differing results, with a larger effect for the syndromic treatment intervention in Mwanza than for the mass treatment intervention in Rakai [28, 29]. Simulation research performed in the early 2000s suggested that population differences in sexual behavior, curable sexually transmitted disease rates, and HIV epidemic stage could explain most of the contrast [30, 31].

Nevertheless, not all phenomena can be explained with traditional epidemic models. Simulation studies can help separate plausible from implausible hypotheses, the evaluation of which can be incorporated into the design of later trials and field studies. For example, multiple randomized controlled trials showed the oral cholera vaccine to be safe and effective. Yet, detailed spatial analysis of the results of cholera vaccine trials in Matlab, Bangladesh, and Kolkata, India, revealed something peculiar, where the strength of indirect effects increased with coverage faster in unvaccinated than in vaccinated individuals [32–34] – a result not yet explained by modeling. In this and similar instances, perhaps further biological understanding will be needed.

## Simulation approaches for infectious disease trial design

**Fig. 2:**
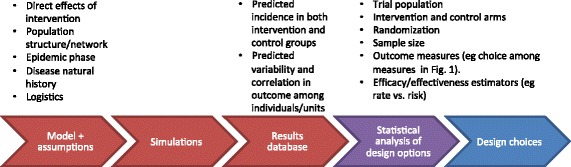
Role of simulations for design and analysis of infectious disease intervention trials. Simulations (*red*) can provide inputs to the usual process of statistical analysis (*purple*) by which considerations of trial population, choice of intervention and control intervention, randomization scheme, estimands and estimators, and sample size lead to a choice of design (*blue*). The simulations take assumptions about the transmission setting of the trial, the individual-level effects of the intervention and the trial itself, and an approach to gathering data from the trial, and create a database of simulated results from many stochastic realizations of the trial. This database contains information on the mean and variability of quantities that would be estimated in the trial under various conditions, which can then inform the design choices

An increasingly popular approach to dealing with the challenges in infectious disease intervention trial design is to employ computer simulations of the trial in the setting of ongoing disease transmission. As illustrated in Fig. 2, such simulations translate assumptions about the effect of an intervention on an individual’s risk, via a mechanistic dynamic model of the disease transmission process and the intervention in the trial setting, into predictions about the magnitude and variability of disease incidence in each trial arm. These simulations explicitly model the process of transmission, including such factors as population and contact network structure, natural history of disease and infectiousness, the phase of the epidemic, assumptions about the direct effects of intervention and, for complex interventions, the timing and logistics of intervention in the trial.

### Simulating a trial

Within this synthetic population, a subset of individuals is selected as a trial population, and simulations of the planned trial are then conducted to generate observations similar to those that could be made in the trial, incorporating the stochasticity, or randomness, inherent in the intervention, the transmission process during the trial, and the observation process. The randomness is generated by figuratively flipping a biased coin in the computer for every possible event. Each single stochastic simulation produces data similar to those which might be observed in one trial. The simulation results can be stored in a database. From each simulated trial, the effect measures of interest can be estimated using a proposed method of statistical data analysis. The mean estimate over the set of simulations under given assumptions allows assessment of whether the trial will provide an unbiased estimate of the quantity of interest, while variability in the estimates produced over a series of simulated trials provides a measure of the likely variability. Sample size and power calculations can be derived directly from the variability of output of many simulations. Alternatively, the intensity and patterns of variability of disease incidence within the simulated trial can be used as inputs to conventional formulas to calculate the effect size that will be detected in the trial and the sample size required to detect such an effect size given the level of variability. By changing the assumptions of the simulation, it is possible to explore the sensitivity of the measured effect size or its variability to particular aspects of trial implementation, and thereby to design more efficient trials. By analyzing simulation results with different statistical methods, it is possible to identify potential biases in the analysis that arise from the transmission process.

### Choice of simulation approach

A key question is what level of detail is required in the simulation. The level of detail can range from full calibration to a country, such as South Africa [35], to simple simulations with dynamics but little explicit demographic structure [12]. Simplicity needs to be balanced with sufficient detail such that the bias in the results is minimized. One needs to carefully weigh each additional complexity and consider whether the choice of intervention and study design is likely to be affected by it. If comparing analytical approaches alone, then purely statistical models may be sufficient [6]. In many contexts, however, dynamic mechanistic models are more useful. In some cases, it may even be important to consider within-host effects.

Simulations for trial design must choose a contact network structure and critically examine that choice because it can affect the predicted power and sample size needed for the trial. Simulations using networks can examine the effect of within-cluster structure on statistical power and sample size [36]. Similarly, they can be used to examine the effect of between-cluster mixing, namely contamination across clusters, on the power of a study and bias of estimates. Incorporating information on network features can improve the efficiency of treatment effect estimation in cluster-randomized trials [37]. In general, simulation of trials on contact networks can aid in understanding the circumstances under which the structures of these networks matter in trial design and when they can be ignored. At a finer level of detail, simulation can help identify network features that are relevant, in particular, the minimal set of such features that still yields a gain in power, and how precisely such features would have to be measured in practice.

The choice of model needs to be matched to the question of interest. Stochastic, individual-based models and deterministic differential equation models can achieve different objectives related to trial design. Stochastic, individual-based models have the advantage of generating data that have statistical variation, and are thus better suited to assess different statistical methods of analysis. Further, they allow greater detail in individual attributes and may also more accurately reproduce the population-level effects to be estimated. Differential equation models generally run much faster and can aid in studying the effects of different combinations of interventions on the outcome, allowing the study of point estimates of effect measures. However, in general, they do not produce synthetic data that can be analyzed using the proposed statistical methods without an additional stochastic layer.

Concern about the robustness of results to model misspecification is often raised. However, advice that emanates from a model may still be qualitatively correct even if the model does not accurately characterize all aspects of the transmission system. For example, the advice in tradeoffs for optimal design in estimating different effects of interventions in two-staged randomized studies [4] is model assisted and is used to draw analytical insights that can be valid even if the model is imperfect.

One challenge to using simulations to design and interpret studies is the difficulty in having the appropriate data to inform all aspects of the simulations, including empirical epidemiological data about the natural history and transmission of the disease or treatment effect. Simulation can assess the impact of uncertainty or lack of knowledge on potentially important epidemic features. An advantage of using simulations after a trial is that a great deal of additional data will be available to inform the modeling. A further challenge to using simulations for trial design is the feasibility of performing such analyses in a timely manner. Often, prior to proposing a trial, funding is not available to develop an appropriate model and conduct simulations for the design. Once funding is available for a study, it is typically necessary for the study to commence soon after by making the best use of conventional sample size calculations. In an emergency, the models and simulations may be performed quickly. However, going forward, efforts must be made to integrate funding of and time for simulations for trial design into the process. In addition to increasing confidence in the choice of population and sample size, the process of designing, implementing, and performing the simulations may provide other benefits, such as insight into potential biases in effect estimates, ways to account for heterogeneities in the trial population, calculations of quantities related to the ethics of trial design, and suggestions in trial design choices beyond sample size that may improve the probability of the trial’s success. Despite potential caveats, the relative cost of computations versus actually running a trial will be very low, while the potential gains in avoiding inconclusive studies are large.

## Conclusions

Expertise in infectious disease dynamics, statistical science, and simulation methods are required to adequately design and interpret trials for many interventions against current and emerging infectious diseases. Trial design should draw in an expert on infectious disease transmission dynamics to work alongside a statistician, who is virtually always employed for study design. Indeed, an increasing number of investigators have both skillsets. While simulation experiments cannot replace trials, most trials would benefit from simulations, or at least from an exercise to signpost the required steps in such simulations that may in itself highlight unexpected aspects of trial design. When planning a trial, it is advantageous to explicitly annotate a timeline of events for each case and consider possible variation in the sequence of these events, similarly to the diagram included in the design of the Ebola ring vaccination trial [7]. Clear communication of complex simulations, of the assumptions underlying them, and their limitations is important. In addition to simulating trials prior to implementation, there is value in validating modeling approaches following the completion of a trial to develop lessons learned for the simulation of future trials. Over the long term, it would be useful to combine simulations and practical experience to develop rules of thumb for adjustment that can provide guides for smaller studies.
